# Native Hawaiian and Pacific Islander populations in genomic research

**DOI:** 10.1038/s41525-024-00428-6

**Published:** 2024-09-30

**Authors:** Edra K. Ha, Daniel Shriner, Shawneequa L. Callier, Lorinda Riley, Adebowale A. Adeyemo, Charles N. Rotimi, Amy R. Bentley

**Affiliations:** 1 Center for Research on Genomics and Global Health, National Human Genome Research Institute, National Institutes of Health, Bethesda, MD USA; 2 University of Hawaiʻi at Mānoa, Honolulu, HI USA; 3 University of Pittsburgh School of Medicine, Pittsburgh, PA USA; 4 Department of Clinical Research and Leadership, The George Washington University School of Medicine and Health Sciences, Washington, DC USA

**Keywords:** Genomics, Genetics research

## Abstract

The role of genomic research and medicine in improving health continues to grow significantly, highlighting the need for increased equitable inclusion of diverse populations in genomics. Native Hawaiian and Pacific Islander (NHPI) communities are often missing from these efforts to ensure that the benefits of genomics are accessible to all individuals. In this article, we analyze the qualities of NHPI populations relevant to their inclusion in genomic research and investigate their current representation using data from the genome-wide association studies (GWAS) catalog. A discussion of the barriers NHPI experience regarding participating in research and recommendations to improve NHPI representation in genomic research are also included.

## Introduction

From clinical care advancement to public health improvement, the many applications of genomic research involve an increasing range of methods and technologies with complex ethical and social implications for both individual and population health^1–3^. Given its far-reaching influences, equitable inclusion of underrepresented populations in genomic research is an important priority, leading to increased calls to diversify the list of populations in which genomic research is conducted^4–6^. Equitable inclusion of diverse populations in genomics extends the applicability of research findings and also ensures that the benefits of genomic research, such as reduced misdiagnosis and more accurate prediction of disease risk, are accessible to all individuals^7–10^.

Some progress has been made towards increased inclusivity in genomic research. The proportion of participants who are not of European descent in the genome-wide association studies (GWAS) catalog increased fivefold from 2009 to 2016. Moreover, in 2019, samples from individuals of African and Hispanic or Latin American ancestry made up 2.03 and 1.13% of individuals included in GWAS, respectively^6,11^. Despite these improvements, representation of Indigenous populations has decreased, with the proportion of Indigenous participants in all GWAS study samples declining from 0.06% in 2009 to 0.02% in 2019^12^. Within the Indigenous grouping are Native Hawaiian and Pacific Islander (NHPI) populations (see Box 1: “What do we mean by NHPI?”), an underrepresented community that has received relatively little attention in genomic research^6,13^. Investigating why and how this underrepresentation is perpetuated in genomic research requires an analysis of the cultural, demographic, and genetic background of NHPI populations. Here, we discuss the unique qualities of NHPI populations, their current representation in genomic research, and barriers to participation in research. We conclude with three recommendations to improve representation of NHPI in genomic research. Given that population descriptors in genomics research are continuously evolving and that genetics does not give biological meaning to race, our use of descriptors for NHPI populations does not and should not be used to suggest that race and ethnicity are biologically meaningful categories for NHPI populations^14^.

Box 1 What do we mean by NHPI? In the context of this paper, NHPI includes individuals whose self-reported ancestry originates from three areas in the Pacific Ocean known as Micronesia (a region consisting of over 2000 small islands that include the Federated States of Micronesia, the Marshall Islands, and Guam), Melanesia (a region south of Micronesia that includes Fiji, Vanuatu, the Solomon Islands, and Papua New Guinea), and Polynesia (the largest, easternmost region that includes Samoa, Tonga, the Hawaiian Islands, and French Polynesia). All three regions are located in the Central and South Pacific Ocean (see map), also known as Oceania, which is why NHPI populations are sometimes referred to as Oceanian^118^. NHPI populations are widely viewed as the Indigenous peoples of the Pacific Rim and are considered to be separate from other populations that are also referred to as Indigenous, such as the Australian Aboriginal peoples and Torres Strait Islanders. Cohorts used in GWAS primarily use self-reports to identify NHPI participants. Our review does not attempt to use genetic data to define any NHPI populations. Image: This image is in the public domain; https://commons.wikimedia.org/wiki/File:Pacific_Culture_Areas.png; author Kahuroa.
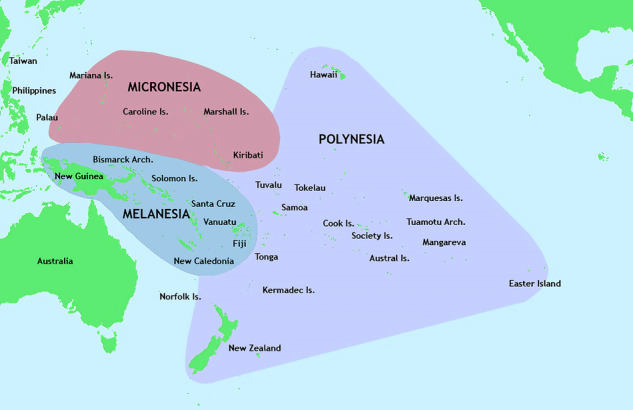

## Cultural and demographic distinctiveness

NHPI populations share a history of colonization, destruction, and historical trauma that are at the root of the present-day health inequities they experience^15^. The impact of historical events on NHPI populations’ health is especially pronounced for Native Hawaiians, who experienced a 90% population decrease following the arrival of Captain James Cook in 1778 and the introduction of infectious diseases such as measles and smallpox^16^. In addition to drastic population decreases, NHPI cultural landscapes were changed by missionaries, whose rhetoric, practice, and enforcement of Christian transformation displaced NHPI customs and traditions^17^. The need to forsake traditional practices and adopt changed appearances and attire was likened to a transformation to allow “good” to conquer “evil.” Ultimately, these influences stripped NHPI communities of their cultures and significantly damaged their physical, mental, and social well-being^18^.

NHPI culture, traditions, and identity are inextricably linked to land. The loss of their land and access to land due to colonization has detrimental physical, mental, social, and psychological consequences on NHPIs^19^. In particular, NHPI populations share a long-standing military presence in their native lands. During World War II, many battles were fought on Pacific Islands resulting in the destruction of their lands and forcing rapid shifts in lifestyle^20,21^. Today, NHPI communities attribute their health condition to these abrupt changes during periods of colonialism and war. Specifically, NHPI populations traditionally consumed a majority of their foods raw or steamed, had access to locally sourced poultry and fish, and consumed a diet high in complex carbohydrates like taro and breadfruit^22,23^. However, years of successive colonization and periods of war led to increased import of refined grains and processed foods such as white rice, high fructose corn syrup products, and red meats, which have slowly replaced traditional NHPI diets due to their availability, affordability, and convenience^24,25^. This replacement of their local diet with canned and instant foods is thought to have catalyzed a diabetogenic diet, and increased barriers to culturally meaningful local forms of physical activity, such as fishing, in their new environment contributed to further metabolic disease^26,27^. Thriving NHPI cultures began to disappear as Western colonization uprooted connections to sources of sustenance and indigenous ways of life^28^.

Another cultural and demographic attribute distinguishing NHPI populations from others is the experience of a unique climate change-induced loss of land and, by extension, cultural identity. Issues such as sea-level rise and king tides—the encroachment of waves on areas that do not usually experience marine influence—reduce the availability of both land and freshwater, contributing to food insecurity and motivating NHPI individuals to migrate away from their homeland^29–32^. The uninhabitability of native lands is also exacerbated by economic factors that further displace NHPI populations and result in a loss of self-identity and an increase in physical and mental health risks^19,33^. For example, Native Hawaiian communities cite the overuse of Native Hawaiian lands due to tourism, identifying how the lack of environmental stewardship continues to damage natural resources that sustain vital elements of their culture and health^34^. Today, more Native Hawaiians live in the continental US than in Hawai’i^35^. Despite these shared experiences between NHPI communities, it is important to note that the NHPI experience is not standardized—NHPI populations are unique, and different island communities experience these forces to varying degrees.

It is a common practice to group NHPI with Asian individuals for demographic purposes (e.g., the Centers for Medicaid and Medicare Services uses Asian Americans/Pacific Islanders (AAPI) as a racial category in research and public reporting of minority health disparities); however, NHPI are distinct from Asian groups^36^. No Asian countries are included among NHPI nations, and no history of unification exists between the two^37^. A resolution creating “Pacific/Asian American Heritage Week” was passed and signed into law in 1978. The 1990 and 2000 censuses included a category of “Asian or Pacific Islander”. The aggregation of these groups was not based on scientific grounds. In 1997, an Office of Management and Budget directive separated “Asian or Pacific Islander” into “Asian”, referring to East, Mainland Southeast Asians, and Island Southeast Asians, and “Native Hawaiian and Other Pacific Islander”^38^. The aggregation/disaggregation of these groups strongly impacts how individuals are recruited into scientific studies and how health outcomes can be evaluated.

NHPI populations’ unique background also extends to the disproportionate burden of disease they experience. Even after adjusting for body mass index (BMI), socioeconomic and lifestyle factors, NHPI individuals experience a higher risk of diseases such as diabetes compared to individuals of European ancestry and the general US population^39,40^. Separating NHPI and Asian populations highlights striking differences in health disparities ranging from cancer mortality rates to risk factors for cardiovascular disease^41,42^. When evaluated as a separate group, NHPI populations consistently experience disproportionate increases in risk factors and outcomes related to a variety of diseases^43,44^. These findings are well-evidenced by past and present research and continue to be attributed to the detrimental effects of colonization that have dismantled NHPI cultural beliefs and practices, barred access to sacred lands, and driven NHPI communities to rural areas with reduced access to transportation, healthy food choices, quality schools, and safe walking paths^45,46^. NHPI individuals are also more likely to report multiple races compared to other racial and ethnic groups^47^. Data aggregation of NHPI populations persists today in an effort to increase statistical power and address issues of generalizability and significance in statistical analyses that arise due to the often small sample sizes of these populations^48,49^. Importantly, consistent demonstration of these disparities during data analysis has prompted NHPI leaders and researchers to advocate for disaggregation of NHPI data^50–53^. We echo this call while acknowledging that the inclusion of larger numbers of NHPI may be needed for meaningful analyses of these individuals in some contexts.

## Genetic clustering

The population genetics characteristics of NHPI populations relative to other population groups also support the need for greater representation of NHPI in genomic research. Importantly, the population history of the islands in this vast region has left identifiable characteristics in the DNA of NHPI individuals. The settling of Remote Oceania is thought to have begun with the migration of Austronesian people from Taiwan and the Philippines ~3000 years ago^54–56^. The primary ancestral component of Polynesians is better represented by Borneons than Han Chinese^57,58^. Papuan ancestry was initially introduced into First Remote Oceanians after the end of the Lapita cultural period and subsequently spread from Near Oceania to Remote Oceania^56,57^. Analysis of genome-wide data on individuals from 21 Pacific Island populations reveals the settling of Polynesia as a succession of founding bottlenecks originating in Samoa and eventually reaching the easternmost Polynesian islands (e.g., Rapa Nui). This migration history is characterized by directional loss of genetic variants along the branching routes in this vast network of islands^59^. Oceanian populations also have relatively small effective population sizes (N_e_), as has been recently estimated for Melanesians^60^ and Samoans^61,62^. Compared with other Human Genome Diversity Project populations, Melanesian individuals were shown to have the among the highest amounts of private^63^, common variation and to have more variation derived from archaic admixture (i.e., Denisovan admixture), differing markedly from East Asians^60^.

**Fig. 1:**
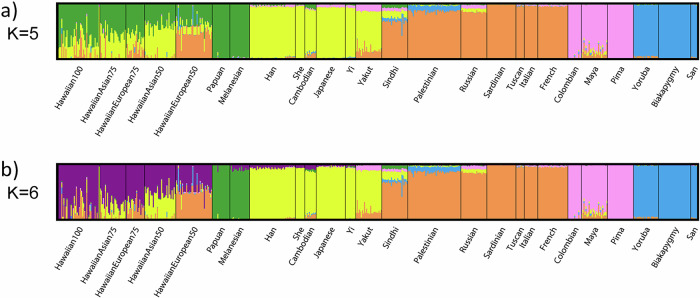
ADMIXTURE clustering of Native Hawaiians. Admixture clustering of Native Hawaiians and HGDP samples. Native Hawaiian ancestry labels, such as HawaiianAsian50, were calculated based on the individual’s report of their parents’ ancestral backgrounds and denote individuals as having 50% Native Hawaiian and 50% Asian ancestry, i.e., one parent with solely Native Hawaiian ancestry and one parent of Asian descent. K refers to a pre-determined number of sub-populations set by the study’s authors, and the colors assigned to these groups were determined through an automated process performed by the ADMIXTURE software program. Panel **a** demonstrates how individuals who self-identify as having NHPI ancestry group separately from other ancestry groups when an analysis is performed with five sub-populations. Panel **b** illustrates how individuals who self-identify as having Native Hawaiian ancestry group separately from other Pacific Islander ancestry groups such as Papuans and Melanesians when analysis is performed with six sub-populations. Reproduced from Kim et al., *PLoS ONE*, 2012 (CC-BY-4.0-https://creativecommons.org/licenses/by/4.0/).

**Fig. 2:**
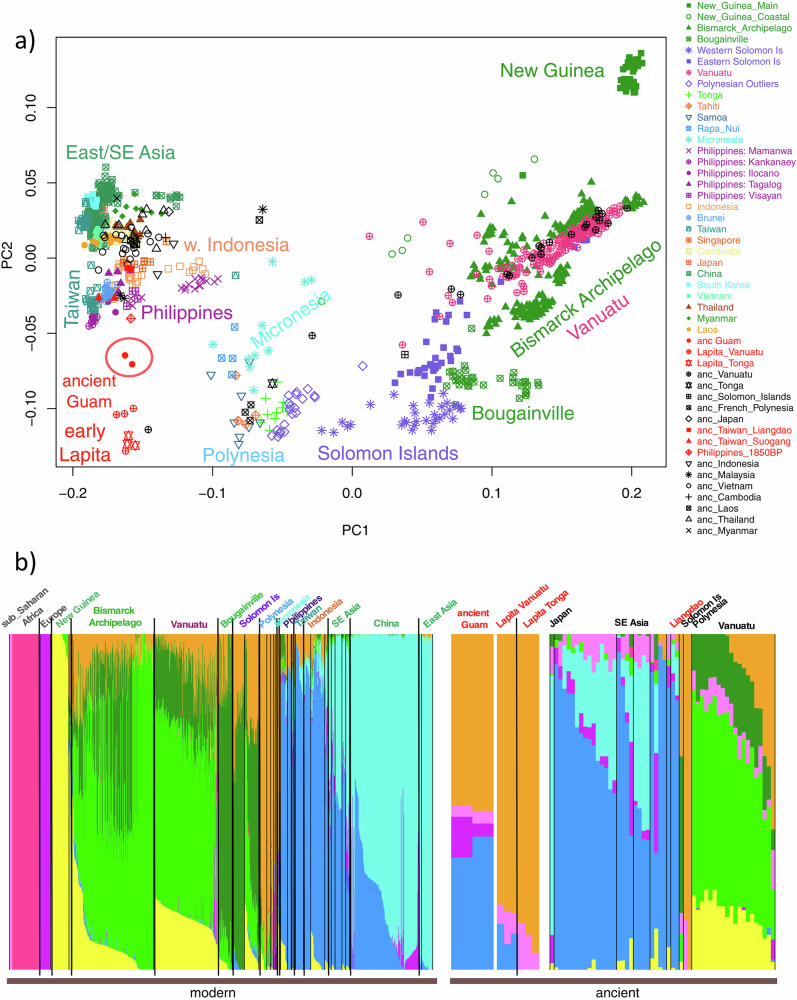
Principal Component (PC) and ADMIXTURE analyses of the ancient Guam samples merged with Human Origins Array data for modern and ancient samples. **a** Plot of the first two PCs. Ancient samples are projected. **b** ADMIXTURE results for K = 9. Population names are color-coded as in the PC plot. Reproduced from Pugach et al., *PNAS*, 2021 (https://creativecommons.org/licenses/by-nc-nd/4.0/).

In addition to founder effects, there is evidence for at least two waves of Papuan admixture into Oceanian individuals, an earlier one that predates the settling of Polynesia and is more uniformly present across Polynesian populations and a more recent one that varies more across Polynesian populations^61^. Early Native American contact with Polynesians has long been speculated due to crops found in Polynesia. Recent genomic analyses suggest pre-Columbian contact between Native Americans (most closely related to present-day indigenous Colombians) and Polynesians^63^. Importantly, Native Hawaiian, Melanesian, Micronesian, and Polynesian samples cluster together and separately from East Asian samples. An admixture plot of NHPI and worldwide samples shows significant admixture among the NHPI, predominantly with individuals of European or East Asian ancestry (Fig. 1a)^50^. Research on sequence identity, as well as the cultural and demographic factors described above, does not support grouping NHPI individuals with Asian Americans, as is common practice in research^64^. There is also considerable genetic diversity among NHPI groups. When a greater number of populations is selected, Papuans and Melanesians cluster separately from Native Hawaiians (Fig. 1b). Melanesians group separately from Native Hawaiians, Ancient Guam, and the Māori^49^ and genetic analysis of Polynesians showed clustering with Micronesians but not Melanesians (Fig. 2)^65^. Importantly, however, the genetic ancestry that seems to define NHPI individuals is not commonly found outside of Oceania^66^. The overall historic isolation and genetic clustering of NHPI populations underline the importance of sufficiently representing NHPI ancestries in genomic research^67^. Aggregation of NHPI samples with other population groups as a result of poor representation may lead to an unappreciated population structure in the data (i.e., subgroups with different allele frequencies), which can lead to spurious findings or to an inability to adequately test for genetic associations that may be best identified in NHPI.

The genetic clustering of NHPI has many implications. As a very well-publicized example, blond-haired individuals from the Solomon Islands arose independently within the Solomon Islands, with a different mode of inheritance than is seen in other instances of the trait worldwide rather than the result of admixture with populations from outside the region^68^. Of clinical significance, NHPI individuals exhibit higher frequencies of some pharmacogenetic variants than better-studied populations, highlighting a need for more pharmacogenomic research^69^. Experiencing elevated risks for adverse outcomes due to the inability of some NHPI to convert pharmaceutical drugs into their active form could be ameliorated with better representation in research^70^. Moreover, having NHPI ancestry is also linked to an increased risk of diseases such as type 2 diabetes and heart failure compared to nearly all other racial/ethnic groups, including non-Hispanic Whites, African-American, and Latino populations in the US, but the genetic factors underlying this observation are unknown^71–73^. The need for better representation of NHPI in genomic research is also apparent in the imputation of genome-wide array data in NHPI, where Native Hawaiians were imputed more poorly than African Americans and Latin Americans^13^. In fact, using imputed data, researchers were unable to detect the strong association signal for *CREBRF* and BMI in Native Hawaiians that was apparent using directly genotyped data^74^.

Attempts to use genetic differences to explain health disparities, such as for obesity, remain prevalent today, with researchers highlighting the need to examine the role of genetic factors to explain NHPI-specific health disparities^40,67,75,76^. In particular, geneticist James Neel’s ‘thrifty genotype’ hypothesis, which posits that positive selection of “thrifty” alleles that promote fat storage was driven by the frequent long ocean voyages taken by Pacific Islanders, is widely used to explain the high prevalence of diabetes and obesity among NHPI individuals^71,77,78^. The once advantageous genotype now predisposes the descendants of these seafaring ancestors to obesity-related conditions and is responsible for the high prevalence of diabetes and obesity among NHPI today^79,80^. The discovery of a Polynesian-specific missense variant in *CREBRF* that is strongly associated with increased BMI in Samoans^81^ and Native Hawaiians^74^ that appeared to be under natural selection was an important discovery, demonstrating the potential for discovery with even modest sample sizes of NHPI. This finding was described as supporting the thrifty gene hypothesis^81^, though this interpretation has been challenged^82^. Critics of the thrifty gene hypothesis have called for its abandonment, citing the lack of conclusive findings, the dissonance between such narratives and cultural history, and overemphasis on genetic predisposition to obesity among these individuals^82–87^. Clearly, there is a need to better represent and involve NHPI populations in research to ensure that stories and theories about genomic variants and how they arose do not precede the function and significance of these variants. Through the better representation of these individuals, racialized genetics can be prevented and replaced with more accurate explanations of NHPI health disparities^84,88^. NHPI’s unique combination of cultural, demographic, environmental, historical, and ancestral histories have important social and genomic implications specific to this population. These factors highlight the critical importance of genomic and other types of biomedical research in NHPI.

## Current NHPI representation in genomic research

Only a few studies on NHPI inclusion in genomic research exist^89^. Continuing to illuminate and monitor the inclusion, or lack thereof, of NHPI is essential for combatting the underrepresentation of NHPI in genomic research. To date, there have been no publications analyzing the representation of NHPI in genome-wide association studies (GWAS), which are a cornerstone of genomic research. To fulfill the need to assess and quantify the current state of NHPI inclusion and representation in genomic research, we conducted an analysis of the GWAS Catalog, a collection of all published GWAS.

**Fig. 3:**
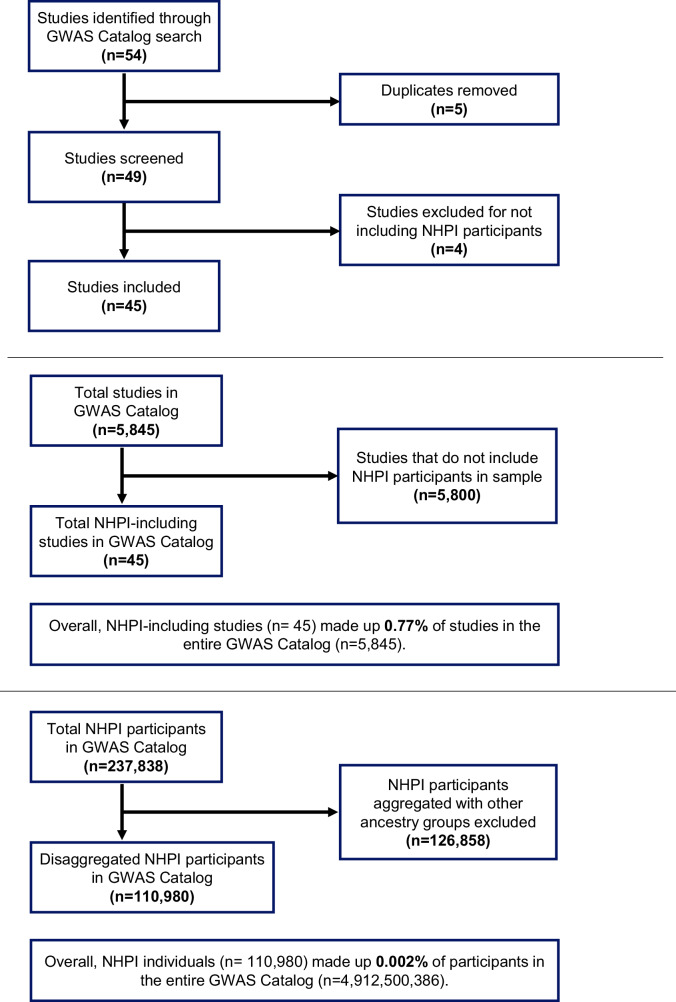
GWAS catalog search flow charts. Flow charts describing the search process for studies that include NHPI participants and the calculation of the proportion of NHPI participants in the GWAS Catalog. The search terms used were “Hawaii,” “Hawaiian,” “Pacific Islander,” “Islander,” “Pacific,” “Oceania,” and “Oceanian.”.

**Fig. 4:**
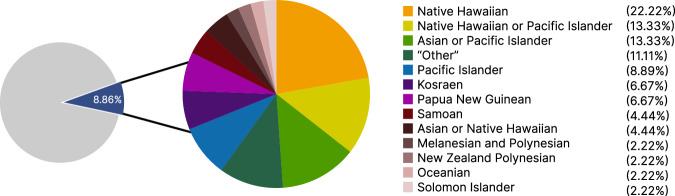
Population descriptors used for NHPI in GWAS studies. Pie chart depicting the percentage of NHPI participants in NHPI-including studies in the GWAS Catalog (*n* = 45) and the population descriptors used to describe NHPI populations.

Using seven search terms informed by the GWAS Catalog’s framework for describing NHPI populations or those with ancestry in the Pacific Islands^90^, a total of 45 studies that included NHPI participants were identified and evaluated with regard to the NHPI population descriptor used, percentage of NHPI in the sample, and outcome(s) of interest (Fig. 3). NHPI individuals made up 0.002% of participants in the entire GWAS Catalog (July 11, 2022). This percentage includes GWAS that utilizes the same cohort(s), resulting in counting individual participants multiple times, with uncertain results for the estimation of NHPI representation in genomic research. There was no consensus in the population descriptors used for NHPI participants, with most studies (47%) having a separate category for the single NHPI population that was studied (e.g., Samoan) or grouping NHPI with Asian ancestry (18%). Of these 45 NHPI-including studies, NHPI participants comprised a small proportion (less than 9%) of the total research participants in the sample (Fig. 4).

The underrepresentation of NHPI Individuals also exists in ongoing efforts to monitor diversity in genomic research. Although the GWAS Diversity Monitor was created to “highlight understudied areas of research”^91^, NHPI participants are either aggregated or missing from this dashboard. In addition, there is no mention of Native Hawaiians, Pacific Islanders, or Oceanian ancestry throughout the website, emphasizing that current efforts to increase diversity have fallen short when it comes to NHPI individuals. Greater attention to NHPI inclusion and representation is needed, which is evidenced not only by their absence in ongoing efforts to monitor and quantify diversity but also in the lack of consensus regarding how to categorize or describe these individuals in genomic research. The difficulty in assessing the genomics of NHPI populations also lies in their aggregation with other populations.

Despite the limitations in the representation of NHPI in genomic research, some notable efforts are worth mentioning. For instance, samples from Melanesia are included in the Human Genome Diversity Project^60,92^, and samples from the Samoan Adiposity Study have been included in the Trans-Omics for Precision Medicine program (TOPMed)^60^, allowing for the harmonized characterization of NHPI samples alongside other worldwide samples. In terms of conducting biomedical research, the Multiethnic Cohort (MEC) study has made the largest contributions to genetic research, including NHPI. MEC is a large epidemiological study recruiting participants from Hawaii and California and includes nearly 4000 Native Hawaiian participants with genetic data. These data have been used to inform the epidemiology of chronic disease traits and diseases^93–96^, particularly cancer^97,98^, consistent with the primary focus of the MEC. MEC data are also included in the Population Architecture using Genomics and Epidemiology (PAGE) Study^99,100^, which focuses on genomic discoveries in diverse populations.

## Barriers to NHPI participation in genomic research

### History of injustice

The historically strained relationship between NHPI and Western nations strongly influences NHPI participation in genomic research today. In the case of the Marshall Islands—a Micronesian nation consisting of multiple islands, on some of which the US military tested nuclear weapons that were a thousand times more powerful than bombs dropped on Hiroshima during World War II—residents on nearby islands were not relocated and suffered from exposure to radioactive fallout^101^. Marshallese distrust in the US was compounded when, over thirty years later, in 1982, the US government approved research to be conducted on the effects of radiation exposure on the Marshallese people^102^. As a result, many Marshallese believe that the US intentionally neglected to relocate inhabitants of nearby islands to increase the number of research subjects^103^. The Marshall Islands now have one of the highest levels of nuclear contamination in the world^104^, and the destruction of entire atolls in the island chain and contamination of the plants and sea life has prevented NHPI populations from returning to their homeland. Similar experiences of unethical research practices, including conducting studies without informed consent and without appropriate language translation, are shared by many NHPI communities, ultimately compounding their distrust in academic researchers, who are often from Western institutions and conduct research from a Western standpoint^105,106^.

Attempts to commercialize NHPI genetic information have been interpreted as an unwelcome imposition of Western property concepts and also undermine their participation in genomic research^107^. In 1995, a patent application submitted by researchers from the National Institutes of Health (NIH) for a virus-infected cell line from a male individual of the Hagahai tribe in Papua New Guinea was approved but formally renounced a year later, as the international community criticized the patent as an act of theft^108,109^. A similar patent application for a cell line originating from the Solomon Islands was withdrawn by the NIH’s National Institute of Neurological Disorders and Stroke^110^. Other NHPI groups, such as the Native Hawaiians, share this experience. When the University of Hawai’i announced a proposal to license the Native Hawaiian genome in 2003, the Native Hawaiian community denounced it as a modern attempt at colonization that would perpetuate colonial damage, especially because the Native Hawaiian people were not consulted and had not given their full, prior and informed consent before the announcement^111–113^. Increasing awareness of how other Indigenous communities have suffered from unethical conduct by researchers, such as the misuse of the Havasupai tribe’s genetic samples, also contributes to NHPI community mistrust in genomic research^20^. This record of ethical violations enshrouding genomic research further marginalizes these groups and discourages their participation in research^114^.

### Misalignment of research and health priorities

Comparing the outcome of interest of NHPI-including GWAS and the findings of the US’ largest in-person household health survey of NHPI populations revealed a potential misalignment of research priorities and NHPI health priorities that may influence NHPI participation in genomic research. Among the 45 studies in the GWAS Catalog that included NHPI participants, cancer, BMI, and schizophrenia were the most studied outcomes. In contrast, the top health concerns for NHPI adults, as identified by the Centers for Disease Control and Prevention’s 2014 Native Hawaiian and Pacific Islander National Health Interview Survey, are diabetes, asthma, and self-assessed general health status^115,116^. Although diabetes and asthma have consistently been identified as health priorities for NHPI populations^117,118^, only two NHPI-including GWAS had diabetes as the outcome of interest, and none studied asthma. This misalignment likely discourages NHPI groups from engaging in research, especially since they have consistently pinpointed focusing on health priorities specific to their communities as a way to encourage their participation in genomic research^89,119^. Thus, aligning genomic research priorities with the burden of disease in NHPI populations is essential to better engage NHPI populations in genomic research.

### Lack of NHPI representation among researchers

Increasing the diversity of participants is linked to increasing the diversity of researchers because engaging in research is contingent on participants’ trust in researchers^120^. A lack of trust in researchers has also been cited as a major barrier to increasing NHPI populations’ willingness to participate in research, highlighting the need for better representation of NHPI among genomic researchers^121–123^. Of NHPI-including GWAS, 21 studies (46.67%) had at least one author with an affiliation with NHPI communities, institutions, or organizations. Interestingly, NHPI-including GWAS with NHPI-affiliated authors had, on average, 638 more NHPI participants in the study sample than NHPI-including GWAS with no NHPI-affiliated authors. This may hint at the potential for increasing NHPI representation in genomic researchers by increasing NHPI representation among researchers who may be better connected to NHPI communities—a finding evidenced in other minoritized groups^34,124^.

Analysis of NHPI representation among researchers in the field of genomics has yet to be published, however, the underrepresentation of NHPI within the medical field is well-evidenced. NHPI representation among faculty in US medical schools remains the same as it was two decades ago, with 85 individuals identifying as NHPI between 2000 and 2020^125^. The role of mentorship is critical—especially for faculty from minoritized populations—for fostering career pathways in academia and research, illustrating how the lack of NHPI faculty contributes to the lack of researchers that encourage NHPI participation and equitable inclusion in genomic research^126^. Disparities in access to research funding may also be affecting the lack of NHPI representation among researchers. The annual funding rate for research proposals submitted to the National Science Foundation by NHPI principal investigators (PIs) is over 11 percentage points below the annual overall relative funding rate for PIs of all racial and ethnic groups, compared to PIs identifying as “Black/African-American” and “Hispanic/Latino” who were 8.1 and 2.1 percentage points below the same annual overall relative funding rate, respectively^127^. Furthermore, funding allocated to Asian American, Native Hawaiian, and Pacific Islander (AA/NHPI) research accounts for 0.2% of total health-related federal expenditures and the overall NIH budget, a number that has remained unchanged for nearly a decade despite these groups being the fastest growing during the same time period^128–130^. The lack of NHPI researchers and disparities in access to NHPI-related research funding are two barriers to increased NHPI participation in genomic research.

## Contributors to NHPI underrepresentation in genomic research

### Continued aggregation of NHPI populations

Continued aggregation of NHPI individuals with Asian Americans and other racial and ethnic groups remains a significant contributor to NHPI underrepresentation today. Although the US Census Bureau officially disaggregated NHPIs for the first time in the year 2000, the Asian American and Pacific Islander (AAPI) and Asian American, Native Hawaiian, and Pacific Islander (AANHPI) categories remain commonly used in research and by state and federal agencies and organizations today, perpetuating a misconception of homogeneity within these populations^131,132^. NHPI individuals are systematically undercounted because they are less likely to fill out census forms on their own, resulting in a net loss of about $1500 per person per year of funding for public health programs that they utilize at higher rates^64^. The subsequent underfunding and lack of NHPI data increase perceptions of structural racism because limited and omitted NHPI data through data collection gaps or inappropriate aggregation of reported data result in under-resourced efforts and policies to support NHPI health, ultimately discouraging their involvement in activities and practices not rooted in NHPI beliefs and culture^133^. As census data influences political representation, resource allocation, and research and policy priorities, the effects of previous aggregation of NHPI populations by the US Census Bureau continues to impact NHPI perceptions of and underrepresentation in research today.

### Variations in NHPI population descriptors

Current genomic research including NHPI individuals exhibits a lack of consensus on how to categorize or describe these groups, creating another challenge in addressing NHPI underrepresentation. Of the 45 studies in the GWAS Catalog that included NHPI participants, the most commonly used NHPI population descriptor was “Native Hawaiian,” which was used in 22% of studies. “Native Hawaiian or Other Pacific Islander” was used in 13% of studies, as was “Asian or Pacific Islander.” NHPI participants were placed into an “Other” category in 11% of studies, with this category always involving aggregation with another group such as American Indian or Alaska Native or with study participants who did not report ethnicity or race. Overall, 56% of current genomic studies including NHPI individuals involved some form of aggregation: 29% of studies aggregated NHPI with other ancestry groups and 27% of studies aggregated multiple NHPI groups together. The aggregation of NHPI remains prevalent, even in genomic research, highlighting the need for greater attention to NHPI inclusion and representation. A lack of consistency in categorizing and describing NHPI individuals in genomic research prevents the scientific community from making progress in achieving true health equity for these populations. A recent positive development is the disaggregation of NHPI participants in the All of Us Research program (allofus.nih.gov) in which “Native Hawaiian or Pacific Islander” is further disaggregated into specific categories, including “Chamorro”, “Chuukese”, “Fijian”, “Marshallese”, “Native Hawaiian”, “Palauan”, “Samoan”, “Tahitian”, “Tongan”, with the option to use free text self-descriptors. These data, however, are not accessible by the public nor by registered researchers who have completed additional security steps to protect participant privacy.

## Future directions

Recent years have seen a rise in the creation and evaluation of ethical guidelines concerning best practices for conducting and promoting genomic research in underrepresented populations. Notable efforts to improve the representation of diverse populations include the Human Heredity and Health in Africa (H3Africa) initiative, which has recruited over 118,000 African participants in 30 African countries to equitably engage in over 51 research projects that are led by African scientists^134,135^. Furthermore, H3Africa continues to expand genomic research capacity in Africa and has significantly contributed to the development of international standards and governance regarding the ethical conduct of genetics and genomics research^136–138^. It is also important to note that existing work to support responsible genomic research among other Indigenous populations, namely the Māori peoples and Aboriginal populations in Canada, are especially relevant in informing improvements to NHPI representation in genomic research. For example, models for biobanks, best practices for Indigenous community engagement, and frameworks for addressing Indigenous representation in genomic research are just a few of the significant contributions of past and present scholars and leaders that can shape initial efforts to improve NHPI representation in genomic research specifically^139–141^. Although many of these frameworks can be applied to improving the underrepresentation of NHPI populations in genomic research, the unique contexts surrounding NHPI groups emphasize the need to develop solutions that combat NHPI-specific barriers to participating in genomic research.

In the context of unique cultural, demographic, colonial/political, and genetic backgrounds, past and present experiences with research, and the current landscape of genomic research, we propose that improving NHPI representation in genomic research requires the ABCs: A is for actualizing NHPI-driven guidance and recommendations, B is for benefit sharing and building trust, and C is for community-based participatory research implementation. Developing these tools of governance ensures fair distribution of the benefits of genomic research and medicine among researchers and populations alike^142^.

### Actualizing NHPI-driven guidance and recommendations

Numerous recommendations and guidance on increasing representation across the full spectrum of genetics and genomic stakeholders have been published by professional organizations and many individual academics and researchers, yet mechanisms and procedures that verify the implementation of and adherence to these guidances and recommendations have yet to be established^143,144^. For example, despite re-consent procedures for reuse of identifiable and anonymized specimens being consistently identified as a priority by NHPI research participants, the US Federal Policy for the Protection of Human Subjects (also known as the Common Rule) does not specifically address re-consent procedures^89,144–146^. For NHPI populations specifically, matters of federal recognition and sovereignty rights for NHPI populations within the US are additional considerations that are likely to impact their participation in research. Furthermore, NHPI populations appear to be divided on these topics, and no consensus exists regarding these issues^147,148^. Although these matters cannot be fully addressed within this article, it is vital for researchers aiming to increase NHPI participation to acknowledge and address the implications of these complex issues on research participation and their significance in influencing NHPI views on genomic research. Actualizing recommendations from NHPI communities better engage these individuals, as well as other stakeholders involved in genomic research, and can help establish clear, specific, and enforceable guidelines from available, existing knowledge that truly improve the representation of NHPI.

### Benefit sharing and building trust

Benefit sharing was defined by Schroeder (2007) as “the action of giving a portion of advantages/profits derived from the use of human genetic resources to the resource providers to achieve justice in exchange, with a particular emphasis on the clear provision of benefits to those who may lack reasonable access to resulting healthcare products and services without providing unethical inducements”^149^. Benefit sharing is a way to build trust with NHPI communities and ensure that their interests are protected. There are currently no benefit sharing mechanisms established in NHPI communities. However, mechanisms can be modeled using existing ones with other marginalized populations: benefit sharing at micro-, meso-, and macro-levels in COVID-19 research has been achieved with African populations^150^, India’s Kani tribe currently receives half of the licensing fees and royalties produced from a drug made from a fruit discovered using their indigenous knowledge^151^, and the Rooibos Benefit Sharing Agreement (RBSA) established in 2019 allowed the indigenous San and Khoi peoples of Southern Africa to receive a share of rooibos profits^152^. Benefit sharing is not limited to financial returns or payments; other mechanisms include improving the capacity for research, specialized skills, or services, and building infrastructure benefitting stakeholders^150^. The principles of benefit sharing also align with existing Indigenous research methodologies centered on the 3 Rs: respect, relationality, and reciprocity^153^.

For NHPI communities specifically, benefit sharing could take the form of subsidized access to drugs and treatment developed using NHPI biospecimens^154^. Additionally, establishing an NHPI-specific biobank led by NHPI community members and researchers could give these populations complete control over their biodata and allow them to genuinely exercise the NHPI agency and expertise. These efforts could be modeled after the Native BioData Consortium, the first non-profit research institute led by Indigenous scientists and tribal members in the US that collects and stores biospecimens of Native American tribal members on sovereign Native American land^155^. It is important to note that genomic research may include findings with limited actionability that are not immediately useful to communities, limiting the development of benefit sharing mechanisms^156^. However, given successes with other marginalized populations, developing benefit sharing approaches and opportunities for genomic research involving NHPI can help build trust and better elucidate solutions to address their underrepresentation.

### Community-based participatory research (CBPR) implementation

Addressing NHPI underrepresentation in genomic research requires taking a collaborative, community-driven approach that prioritizes Indigenous agency and expertise to ensure that NHPI participants sufficiently trust and understand the research in which they may be involved. As a research approach that fosters reciprocity between community and academic partners and recognizes that community members are active, equal partners in determining the research agenda, process, and results, CBPR has been shown to be especially successful in engaging NHPI in research^157^. Though CBPR has not been widely implemented in the fields of genetics and genomics, it is considered well-suited to support diversity in genomics and build the trust that underrepresented communities need to engage in genomic research^158^. Additionally, a genetics study aiming to recruit NHPI individuals using a CBPR approach yielded a recruitment rate of over 95%, evidencing the effectiveness of CPBR implementation in efforts to address NHPI underrepresentation^159^. Misalignment of research and health priorities can also be directly addressed through CBPR since NHPI communities can identify health concerns of practical significance to investigate through genomic research^159^. Using a CBPR approach involves a relationship between researchers and participants that is developed through shared interests over time, allowing research questions to be identified by or in collaboration with a community and is driven by the community’s needs^159^. In the context of NHPI representation in genomic research, increased implementation of CBPR can help NHPI communities to directly develop research questions, define the health burdens to be studied and addressed, and design the parameters for their participation in and priorities of the genomic research in which they choose to be involved.

## Summary

Analysis of the GWAS Catalog revealed an underrepresentation of NHPI in genomic research, as evidenced by the low proportion of NHPI participants and NHPI-including GWAS, lack of consensus on how to categorize or describe NHPI groups, and misalignment of genomic research priorities compared to the burden of disease in NHPI communities. This underrepresentation highlights the need to acknowledge, analyze, and address past, present, and potential barriers faced by these communities, including a long history of injustice and continued aggregation of NHPI groups with other racial and ethnic groups. It is vital to include and consider the cultural and historical influences on populations like the NHPI when it comes to equitable inclusion of underrepresented populations in genomic research^142^. Previous studies have highlighted these same discrepancies in current genomic research cohorts that emphasize the diversity of their catalog, yet NHPI representation is limited to data from a single NHPI individual in the study sample^13^. Although efforts to create and evaluate best practices for conducting and promoting ethical genomic research in underrepresented populations have significantly increased in recent years, developing solutions specific to NHPI are needed in light of their cultural, demographic, and genetic backgrounds. Building and implementing systems that actualize guidance and recommendations provided by NHPI communities in regard to conducting research, promoting, and prioritizing the re-building of trust and investigating what continues to prevent NHPI participation through collaborative, community-driven approaches are promising ways to improve the underrepresentation of NHPI and truly bring the benefits of genomic research to all.

## Data Availability

Data used in the manuscript is freely accessible in the GWAS catalog (https://www.ebi.ac.uk/gwas/). Spreadsheets used to describe these data are available from the authors upon reasonable request.
